# Effect of lanthanum carbonate and calcium acetate in the treatment of hyperphosphatemia in patients of chronic kidney disease

**DOI:** 10.4103/0253-7613.56074

**Published:** 2009-08

**Authors:** P. Thomas Scaria, Reneega Gangadhar, Ramdas Pisharody

**Affiliations:** Department of Pharmacology and Therapeutics, Government Medical College, Thiruvananthapuram, India; 1 Department of Pharmacology, Government Medical College, Kottayam, India; 2 Department of Nephrology, Government Medical College, Thiruvananthapuram, Kerala, India

**Keywords:** Cross-over study, hyperphosphatemia, phosphate binders, lanthanum carbonate, serum phosphorous

## Abstract

**Objectives:**

The tolerability and efficacy of lanthanum carbonate has not been studied in the Indian population. This study was, therefore, undertaken to compare the efficacy and tolerability of lanthanum carbonate with calcium acetate in patients with stage 4 chronic kidney disease.

**Design:**

A randomized open label two group cross-over study.

**Materials and Methods:**

Following Institutional Ethics Committee approval and valid consent, patients with stage 4 chronic kidney disease were randomized to receive either lanthanum carbonate 500mg thrice daily or calcium acetate 667 mg thrice daily for 4 weeks. After a 4-week washout period, the patients were crossed over for another 4 weeks. Serum phosphorous, serum calcium, serum alkaline phosphatase, and serum creatinine were estimated at fixed intervals.

**Results:**

Twenty-six patients were enrolled in the study. The mean serum phosphorous concentrations showed a declining trend with lanthanum carbonate (from pre-drug levels of 7.88 ± 1.52 mg/dL-7.14 ± 1.51 mg/dL) and calcium acetate (from pre-drug levels of 7.54 ± 1.39 mg/dL-6.51 ± 1.38 mg/dL). A statistically significant difference was seen when comparing the change in serum calcium produced by these drugs (*P* < 0.05). Serum calcium levels increased with calcium acetate (from pre-drug levels of 7.01 ± 1.07-7.46 ± 0.74 mg dL), while it decreased with lanthanum carbonate (from pre-drug levels 7.43 ± 0.77-7.14 ± 0.72 mg/dL). The incidence of adverse effects was greater with lanthanum carbonate.

**Conclusion:**

Lanthanum carbonate and calcium acetate are equally effective phosphate binders with trends obvious in the first 4 weeks of therapy. The decrease in serum calcium levels with lanthanum carbonate when compared to the increase in serum calcium levels due to calcium acetate is statistically significant. The drawback of lanthanum carbonate is its high cost and relatively higher incidence of adverse events during treatment.

## Introduction

Hyperphosphatemia is a universal complication in patients with chronic kidney disease (CKD) when the glomerular filtration rate (GFR) falls below 25 ml/min. It is now known to be associated with the development of hyperparathyroidism, renal osteodystrophy, metastatic, and vascular calcification resulting in increased morbidity and mortality.[1–3] Elevated calcium × phosphorous product and increased daily intake of calcium are other risk factors for coronary artery calcification.[4] The reduction of serum phosphorus levels in patients with CKD can be achieved through a combination of dietary control, removal by dialysis, and intervention with phosphate binders that prevent the absorption of phosphorus from the intestine. Dietary restriction is not possible beyond a point without risking a negative nitrogen balance.[5] Even regular daily dialysis treatment removes 75% of absorbed phosphorus and not all of the phosphorus ingested.[6] As a result, most CKD patients require phosphate binders to decrease phosphorus absorption from the gastrointestinal tract and maintain the serum phosphorous and calcium × phosphorous product at the recommended levels.

Aluminium-based phosphate binders are not preferred due to associated toxicities like encephalopathy, microcytic anemia, osteomalacia, and arthropathy.[7,8] The calcium salts if given with inappropriate calcitriol treatment can cause hypercalcemia and potentially lead to cardiovascular calcification.[4] It has been observed that long-term, calcium-based phosphate binder therapy can lead to progressive calcification of both coronary and aortic arteries when compared with non-calcium-based agents.[9] Despite these drawbacks, calcium salts especially calcium acetate is the most commonly used phosphate binder due to its efficacy and low cost. The problems associated with the use of aluminium- and calcium-based agents have prompted researchers to develop alternative phosphate binders not containing aluminium or calcium.

Lanthanum carbonate (La_2_[CO_3_]_3_), is a recently developed phosphate binder which does not contain aluminium or calcium.[10] Lanthanum (La) is a naturally occurring rare earth element (atomic number: 57) that can be detected in the tissues of healthy individuals.[11] Lanthanum carbonate acts by dissociating in the acid environment of the upper gastrointestinal tract to release lanthanum ions that bind dietary phosphate released from food during digestion to form lanthanum phosphate, an insoluble compound that is poorly absorbed across the gut wall.[12] Lanthanum carbonate is well tolerated and the adverse effects were mainly gastrointestinal like nausea and vomiting.[13,14] The present study was undertaken to compare the efficacy and tolerability of lanthanum carbonate with the conventional phosphate binder, calcium acetate in our patient population.

## Materials and Methods

This was a randomized open label two-group cross-over study initiated after obtaining Institutional Ethics Committee approval. Patients aged between 18 and 80 years with stage 4 CKD, having serum phosphorous above 5.5 mg/dL, and without significant hypercalcemia or hypocalcemia (serum calcium > 11 mg/dL or < 7.9 mg/dL) were enrolled in the study after obtaining written informed consent. Patients with end-stage renal disease, extensive edema and hypoproteinemia, allergy to any of the medications, or had acute on chronic renal failure were excluded from the study. Pregnant or lactating women, or who were not using appropriate birth control were also excluded. Further, patients with severe gastrointestinal symptoms precluding administration of drugs or gastrointestinal bleeding, malignancy, or exposure to other investigational drugs within 30 days prior to the start of the study were also excluded. Consumption of medications containing calcium, phosphorous, aluminium, or magnesium was not allowed during the study period. Vitamin D supplementation was allowed provided the dose was kept constant during the study period.

The total duration of the study for each patient was of 12 weeks, which could be divided into two stages of 4 weeks each with a 4-week washout period in between. The patients meeting the inclusion criteria were randomly allocated into two groups, by using random numbers obtained from a random number table, to receive either lanthanum carbonate or calcium acetate for 4 weeks. At the end of the first stage, the respective drug was stopped and the patient entered a 4-week drug-free washout period. After the 4-week washout period, the patients were crossed over to receive the alternate drug for 4 weeks. The patients served as their own controls. The patients were assessed at 4 weekly intervals i.e. at the time of recruitment, after first stage (at 4th week), after washout (at 8^th^ week), and after second stage (at 12^th^ week). A detailed history, general examination, vital signs, and relevant systemic examination were carried out at the time of recruitment and at each visit. Serum phosphorous, serum calcium, serum alkaline phosphatase and serum creatinine were estimated at baseline and at each visit.

Calcium acetate 667 mg tablet was administered thrice daily (667 mg of calcium acetate is equivalent to 169 mg of elemental calcium). It was swallowed along with the meals or immediately after the meals. Lanthanum carbonate 500 mg was also taken thrice daily and the tablet was chewed along with the meals.

All data were recorded in a specific case record form (CRF) designed for this study. Compliance was estimated by pill count. The drug-related adverse events that occurred during the study period also were noted in the CRF. The costs of 4-week treatments with lanthanum carbonate and calcium acetate were calculated from the M.R.P. labels on the respective packages.

### Statistical considerations

The sample size was calculated using the power analysis and sample size (PASS 2005) software. A sample size of 20 would ensure that a two-sided test has 80% power to detect a mean difference of 2 mg/dL between the two treatments. Data were fed into the statistical package 70 of Microsoft Excel and checked for data entry errors. The distribution of variables was noted. Data from the patients who completed the study were analyzed using SPSS software. For comparison of the means between the groups, paired *t*-test was used. The level of significance was fixed at 5%.

## Results

**Figure 1:**
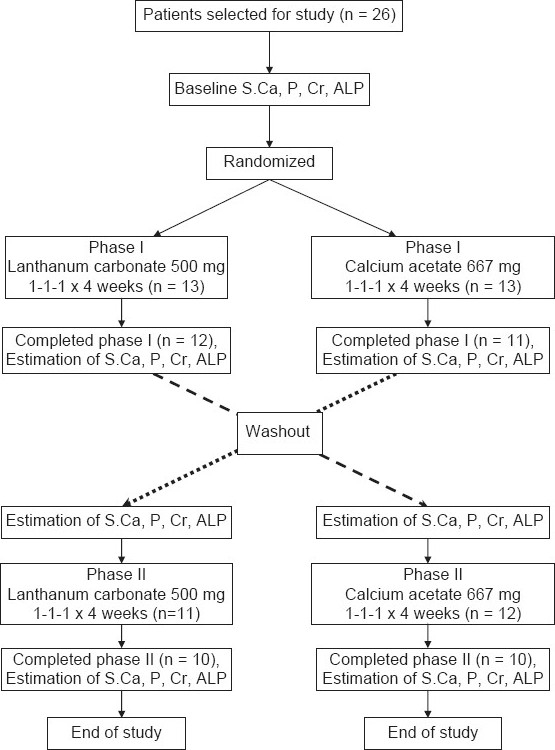
Study flowchart

Out of 26 patients enrolled for the study, 20 patients completed the study [Figure 1]. Of the six patients who did not complete the study, one patient withdrew during phase I (pre-washout) due to adverse effects while on lanthanum carbonate; two patients were withdrawn in phase I due to protocol violation while on calcium acetate; two patients were withdrawn in phase II (post-washout) due to worsening of their renal status while on lanthanum, and one patient was withdrawn in phase II due to worsening of renal status while on calcium acetate. The compliance of the patients during the study was found to be more than 90%. The demographic details of the patients who completed the study are shown in Table 1.

**Table 1:** Demographic data of patients

*Feature*	*Treated population*
No. of patients	20
Mean age (years)	49.9
Mean weight (kg)	55.5
Gender (% male)	65

**Figure 2:**
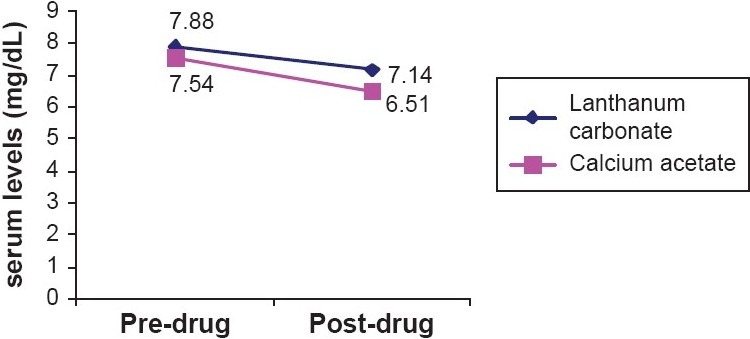
Changes in S. phosphorous levels during treatment

The mean serum phosphorous concentrations showed a declining trend during the period in which the phosphate binders were taken. The mean serum phosphorous levels during the intake of lanthanum carbonate decreased from 7.88 ± 1.52 mg/ dL-7.14 ± 1.51 mg/dL, while during calcium acetate treatment it decreased from 7.54 ± 1.39 mg/dL-6.51 ± 1.38 mg/ dL. The reduction in serum phosphorous produced by lanthanum carbonate and calcium acetate was identical [Figure 2].

**Figure 3:**
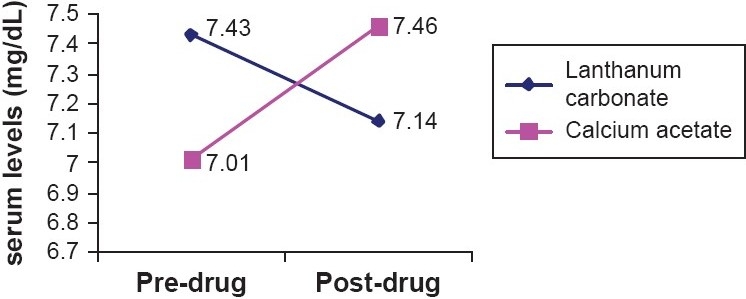
Changes in S. calcium levels during treatment

The changes in mean serum calcium levels during treatment with lanthanum carbonate and calcium acetate showed opposing trends. Serum calcium increased from 7.01 ± 1.07-7.46 ± 0.74 mg/dL with calcium acetate treatment, while it decreased from 7.43 ± 0.77-7.14 ± 0.72 mg/dL with lanthanum carbonate. A statistically significant difference was seen when comparing the change in serum calcium produced by these drugs (*P* < 0.05) [Figure 3].

**Figure 4:**
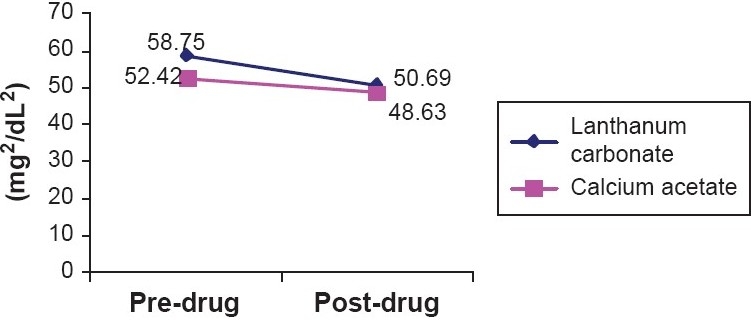
Changes in Ca × P during treament

The mean calcium × phosphorous product showed a declining trend during treatment with both the phosphate binders. The mean Ca × P product during treatment with lanthanum carbonate decreased from 58.75 ± 13.93 to 50.69 ± 10.15 mg^2^/dL^2^ and during treatment with calcium acetate decreased from 52.42 ± 10.32 to 48.63 ± 11.63 mg^2^/dL^2^ [Figure 4].

The changes in alkaline phosphatase levels during treatment with either of the phosphate binders and the difference in the trends of alkaline phosphatase during treatment were not statistically significant. The mean creatinine levels were stable throughout the treatment [Table 2].

**Table 2:** Comparison of laboratory parameters in patients treated with lanthanum carbonate and calcium acetate (n=20)

*Parameter*	*Drug*	*N*	*Pre-drug mean (SD)*	*Post-drug mean (SD)*
Phosphorous (mg/dL)	Lanthanum carbonate	20	7.88 (1.52)	7.14 (1.51)
	Calcium acetate	20	7.54 (1.39)	6.51 (1.38)
Calcium (mg/dL)	Lanthanum carbonate	20	7.43 (0.77)*	7.14 (0.72)*
	Calcium acetate	20	7.01 (1.07)*	7.46 (0.74)*
Calcium phosphorous product (mg^2^/dL^2^)	Lanthanum carbonate	20	58.75 (13.93)	50.69 (10.15)
	Calcium acetate	20	52.42 (10.32)	48.63 (11.63)
Alkaline phosphatase (IU/L)	Lanthanum carbonate	20	216.10 (25.45)	226.65 (71.30)
	Calcium acetate	20	205.90 (37.25)	209.55 (30.91)
Creatinine (mg/dL)	Lanthanum carbonate	20	4.82 (1.69)	5.04 (1.96)
	Calcium acetate	20	4.85 (1.94)	4.86 (2.02)

* The difference in serum calcium levels produced by calcium acetate as compared to lanthanum carbonate was statistically significant (*P* < 0.05)

### Adverse events

Over the entire course of the study, four adverse events were reported by patients on lanthanum carbonate including abdominal discomfort, burning sensation all over the body and vomiting. One patient was withdrawn from the study due to generalized rashes and small eruptions in the oral mucosa. No adverse event was reported during the intake of calcium acetate.

### Comparative costs of treatment

The cost of a single tablet of lanthanum carbonate was Rs.16, resulting into total cost of Rs. 1344 for 4-week treatment. On the other hand, the cost of a 4-week treatment with calcium acetate was Rs.79.80.s The cost of treatment with lanthanum carbonate is 16 times that of the conventional drug, i.e. calcium acetate.

## Discussion

In this study, the efficacy of the newer non-calcium, non-aluminum phosphate binder i.e. lanthanum carbonate was compared with the conventional calcium-containing phosphate binder, calcium acetate, by measuring the changes in the levels of serum phosphorus during treatment with either drugs. It was observed that both drugs were equally good phosphate binders and lower phosphate levels. As both these drugs were given at a fixed dose and only for a limited period (4 weeks), no statistically significant difference was noted in the phosphate-binding ability of the drugs. Also, the phosphate levels could not be lowered to the desirable levels in the majority of patients due to the above-mentioned reasons. It seems that adequate control of phosphate levels may be achieved by using doses considerably lower than that prescribed for the western population especially in the case of calcium acetate. A treat-to-goal approach, in which the doses are titrated upward at two weekly intervals till phosphate levels are controlled, is needed to definitely identify a difference in the efficacy of these phosphate binders, if at all they exist.

Serum calcium needs to be maintained at high normal levels (9-10 mg/dL) and Ca × P product needs to be kept under 55 mg^2^/dL^2^ in patients with CKD. When these parameters rise above the levels specified, there is a higher incidence of vascular calcification and cardiovascular morbidity.[4] Even with the limited data obtained from the study, it could be inferred that the usage of calcium acetate as a phosphate binder was associated with a statistically significant rise in serum calcium when compared to the fall in serum calcium produced by lanthanum carbonate. The difference in the fall in Ca × P product during treatment with the two drugs was not statistically significant. Still the trend of decline in Ca × P product was greater with lanthanum carbonate when compared to calcium acetate.

The level of alkaline phosphatase is an indicator of osteoblast activity. Usually, in CKD, there is hyperphosphatemia and hypocalcemia that produces hyperparathyroidism, which in turn leads to high-turnover bone disease. In such a situation, the alkaline phosphatase levels rise. Since the alkaline phosphatase levels remained fairly uniform throughout the treatment period of both drugs, it may be reasonably assumed that the ability of both these drugs to retard the development of high-turnover bone disease is similar. Further studies are needed, before this assumption is confirmed.

One criterion that needs to be satisfied in the case of a crossover study is that the patient should be having a chronic disease and the condition of the patient should remain stable throughout the course of the study. The steady creatinine values observed during the study confirm the stable condition of the 20 patients who completed the study.

There are no studies comparing the efficacy of lanthanum carbonate and calcium acetate. The only trial that has been conducted comparing lanthanum carbonate with a calcium salt (calcium carbonate) by Hutchison *et al.* concluded that serum phosphate control with lanthanum carbonate (750-3000 mg/day) is similar to that seen with calcium carbonate (1500-9000 mg/day), but with a significantly reduced incidence of hypercalcemia.[14] It is also mentioned that lanthanum carbonate is well tolerated and may be more effective in reducing calcium × phosphorous product than calcium carbonate. These conclusions are similar to the inferences drawn from the present study.

After evaluating the adverse event profile of the drugs, it can be seen that calcium acetate is better tolerated than lanthanum carbonate. There were no adverse events when the patients were on calcium acetate, but on lanthanum carbonate, four patients had gastrointestinal adverse events and one patient was withdrawn due to generalized rashes and small eruptions in the oral mucosa. A placebo controlled, double blind study by Al-Baaj *et al.*, had also observed that gastrointestinal adverse events were predominant with lanthanum carbonate.[15]

In a developing country like India, the cost of treatment is a very significant factor influencing treatment. Affordability of the prescribed treatment significantly influences compliance. On calculating the cost of a comparable dosage regimen of lanthanum carbonate and calcium acetate, it was seen that the former is approximately 16 times costlier than the latter. The high cost associated with lanthanum carbonate treatment makes it beyond the reach of a good proportion of our population.

### Limitations of the study

The study drugs were administered for a limited duration and at a fixed dose. Ideally, the dose should be titrated every 2 weeks for each patient and then the optimal dose has to be maintained for 4-6 weeks or higher. This format was not chosen, as this would put a higher financial burden on the patient due to frequent visits and result in higher dropouts. Dietary phosphorous intake may be different for different patients, as no uniform diet was prescribed due to the difficulty in confirming the patients' adherence to the diet. Another limitation of this study was that it was not blinded. The main reason for this was that the parameters of concern were lab values. Also, since the comparison was between two active compounds, the “double dummy” method had to be used and this was not practically feasible. Selection bias was not a major problem as each patient was receiving lanthanum carbonate and calcium acetate.

## Conclusion

Lanthanum carbonate and calcium acetate are equally effective phosphate binders with trends obvious in the first 4 weeks of therapy. The changes associated with serum calcium levels with both the drugs were statistically significant. The drawback of lanthanum carbonate is its high cost and relatively higher incidence of adverse events during treatment. If both the serum calcium and phosphorous levels are elevated, lanthanum carbonate can be preferred over calcium acetate if the patient can afford it. If there is hyperphosphatemia with low-normal or low serum calcium levels, calcium acetate is preferred. The selection of phosphate binders for the treatment of hyperphosphatemia in CKD should be made keeping in mind the serum phosphorous, serum calcium, the calcium × phosphorous product, and the socioeconomic status of the patient.

Further treat to goal studies are needed to establish whether there is a significant difference in efficacy between the two phosphate binders.
